# Conversion from environmental filtering to randomness as assembly rule of ground beetle assemblages along an urbanization gradient

**DOI:** 10.1038/s41598-018-35293-8

**Published:** 2018-11-19

**Authors:** Tibor Magura, Gábor L. Lövei, Béla Tóthmérész

**Affiliations:** 1 Department of Ecology, University of Debrecen, Debrecen, Hungary; 2 Department of Agroecology, Aarhus University, Flakkebjerg Research Centre, Slagelse, Denmark; 3 MTA-DE Biodiversity and Ecosystem Services Research Group, University of Debrecen, Debrecen, Hungary

## Abstract

Urbanization fragments, isolates or eliminates natural habitats, and changes the structure and composition of assemblages living in the remaining natural fragments. Knowing assembly rules is necessary to support and/or maintain biodiversity in urban habitats. We hypothesized that forest communities in rural sites are organized by environmental filtering, but this may be changed by urbanization, and in the suburban and urban forest fragments replaced by randomly organized assemblages, influenced by the colonization of species from the surrounding matrix. Evaluating simultaneously the functional and phylogenetic relationships of co-existing species, we showed that at the rural sites, co-existing ground beetle species were functionally and phylogenetically more similar than expected by chance, indicating that environmental filtering was the likely process structuring these communities. Contrary to this, in urban and suburban sites, the co-occurring species were functionally and phylogenetically not different from the null model, indicating randomly structured assemblages. According to our findings, changes in environmental and habitat characteristics accompanied by urbanization lead to assemblages of randomly colonized species from the surrounding matrix, threatening proper ecosystem functioning. To reassemble stochastically assembled species of urban and suburban fragments to structured, properly functioning communities, appropriate management strategies are needed which simultaneously consider recreational, economic and conservation criteria.

## Introduction

Anthropogenic activities, like farming, forestry and urbanization are increasing worldwide, and cause significant changes to biodiversity^1^. Urbanization is steadily advancing: today, 54% of the global human population lives in urbanized areas, and this trend is projected to continue in the following decades^2,3^.

Urbanization alters and modifies natural habitats; they are becoming fragmented or even destroyed^4^. The fragmented natural habitat patches in urbanized areas frequently become isolated from each other, making the between-patch dispersal of species and the nutrient flows between the patches difficult or even impossible^5^. Urbanization involves profound modification in deposition of pollutants^6^, in environmental parameters (temperature^7^, humidity^8^), in the amount of nutrients (carbon^9^, nitrogen^10^), and in several biological processes (e.g. leaf -litter decomposition^10^, gene flow^5^). Ultimately, these modifications cause changes in the structure and composition of biotic communities^11,12^.

Understanding how urbanization impacts wildlife is crucial to conserving biodiversity^13^. Earlier papers addressing the effects of changes in environmental and habitat parameters accompanied by urbanization on biotic communities have usually evaluated the abundance and/or taxonomic diversity (species richness and/or species diversity)^14^. Abundance and taxonomic diversity, however, contain little information on the mechanisms influencing patterns of community assembly, although such information is essential in biodiversity conservation^15^.

During the evaluation of community assembly mechanisms, differences (or similarities) in species characteristics are crucial aspects, because processes act on the similarities and differences of the constituting organisms^16^. Functional and phylogenetic characteristics are necessary to quantify such differences, because functionally similar species usually utilise the same resources, while phylogenetically related species may share many morphological and ecological traits through their common origin and evolutionary history^17^. Therefore, incorporating the functional and phylogenetic relatedness of species in a community may enhance our understanding of the mechanisms that create the studied community^15,18–20^. Several recent urban studies tried to deepen such understanding using functional^21,22^ or phylogenetic approaches^23,24^.

Ecological communities at local scales are assembled from the regional species pool according to deterministic (habitat/environmental filtering, ecological/species interactions) or stochastic (random) processes^25,26^. During environmental filtering, species with appropriate traits and tolerance limits for the target environment can persist^27^, while species that lack those traits or tolerance limits are filtered out^25^. Environmental filtering operating on conserved traits results in functional and phylogenetic clustering (under-dispersion), when co-occurring species form assemblages that are functionally and phylogenetically more similar than predicted by chance^17,26^. Environmental filters working on convergent traits, on the other hand, cause functional clustering, but phylogenetic repulsion (over-dispersion), since the co-existing species will be functionally more, but phylogenetically less similar than predicted by chance^17^. If biotic (species) interactions (competition, facilitation) are the main forces structuring the assemblage, ecologically similar species that occupy the same niche and/or habitat will be more rare than random. Species interactions acting on conserved traits produce functional and phylogenetic repulsion: closely related species with similar traits will be excluded, and co-existing species will be functionally and phylogenetically less similar than predicted by chance^17^. Species interactions operating on convergent traits, on the other hand, cause functional repulsion, but random phylogeny: co-existing species will be functionally less similar than predicted by chance, but their phylogenetic relatedness will be random^17^. If community assembly is driven by random processes, co-occurring species will be functionally or/and phylogenetically not different from randomly generated “null” assemblages^17,28^.

Although understanding the assembly rules determining local community organization from regional pool represents a fundamental topic in ecology and conservation biology^29^, the mechanisms and underlying patterns of community assembly along urbanization gradients are rarely studied^24^. Such analyses on invertebrates seem to be missing. Our study aimed to assess the assembly processes underlying the coexistence of ground beetles (Coleoptera: Carabidae) across an urbanization gradient in Hungary.

Ground beetles are highly appropriate objects for such a study, because they are taxonomically well known, common in most terrestrial habitats, can easily be collected using standard methods^30^, often are used as indicators of environmental quality^31^, and several congeners frequently co-occur, making them a suitable group to explore community assembly mechanisms^20^. Several previous papers have evaluated community composition and coexistence patterns of ground beetles along urbanization gradient^32–35^, but none of these included a functional and/or phylogenetic analysis. Using functional and phylogenetic characteristics, we aimed at to assess whether ground beetles are deterministically or stochastically structured along the rural-suburban-urban forest gradient representing increasing intensities of human disturbance. Previous studies in the same region^33,36^ showed that ground beetle species composition of the forested rural sites was different from that of the forest remnants at the suburban and urban sites due to the species flow (spillover) from the surrounding matrix into these latter sites. Therefore, we hypothesized (H1) that specific environmental conditions in the rural forest sites (closed canopy, cool and moist microclimate, high amount of coarse woody debris) would constitute strong environmental filtering, and select related species with specific traits that allow to cope with these specific habitat conditions. Consequently, co-occurring species in the rural sites would be functionally and phylogenetically more similar than predicted by chance (functional and phylogenetic clustering). Contrary, we expected (H2) that in the suburban and urban sites, due to the unpredictable, random species flow from the surrounding matrix^36^, ground beetle communities would be stochastically structured and thus co-occurring species would not be different, either functionally or phylogenetically, from random assemblages.

In the present study, we found that ground beetle communities organized previously by environmental filtering cannot remain intact by urbanization, and are replaced by randomly organised assemblages, influenced by colonization of species from the surrounding matrix in suburban and urban habitats.

## Materials and Methods

### Study area and sampling design

An urbanization gradient was designated from an extensive lowland forest, near the eastern Hungarian city of Debrecen (47°32′N; 21°38′E) towards the suburban areas, and the centre of the city. Debrecen is the second largest city of Hungary (203 283 inhabitants in 2015), and located on the eastern plains near the country’s eastern border^33^. Along the studied urbanization gradient three forested areas, representing rural, suburban and urban habitats, were selected. These areas belong to a once-continuous old forest stand (>100 years) dominated by English oak (*Quercus robur*), and recently are embedded within an agricultural matrix with undisturbed or moderately disturbed habitats (e.g., meadows, grasslands, pastures, agricultural areas). The present area of the forest is 1 082 ha, and in spite of its contraction as the city grew, still large enough to allow numerous forest-associated plant and animal species to maintain self-supporting populations. The urbanization level of the areas was characterized by the relative built-up area, measured by the ArcGIS program using aerial photographs. In the rural, continuous forest there was no built-up area. In the suburban zone, approximately 30% of the surface was built-up or paved, while in the urban area, 60% of the surface was built-up or drastically different from the original forest habitat. Areas were also varied in additional (not quantified) types of disturbance, such as the presence of people and the frequency and intensity of habitat management/maintenance operations. In the rural forest there was no regular forestry intervention, and the presence of people was minimal. In the suburban area, the fallen trees and branches were regularly removed, and the level of disturbance from human visitors was moderate. In the urban area, the fallen branches and decaying trees were also regularly removed, and the shrub layer was strongly thinned. Paths were covered with asphalt, and human disturbance was considerable. Sampling areas (rural, suburban, urban) were about 1–3 km distant from each other^33,36^.

Within each of the three sampling areas, four sites, at least 100–150 m from each other were selected. Ground beetles were collected at each of these sites using pitfall traps. During the sampling, we followed Niemelä *et al*.^37^, who proposed that pitfall traps should be installed in a random arrangement at least 10 m apart to ensure independent sampling. This resulted in a total of 120 traps (3 areas × 4 sites × 10 traps). Each pitfall trap was at least 50 m from the nearest forest edge in order to avoid edge effects^38^. Pitfall traps were plastic cups (diameter 65 mm) containing about 100 ml of 75% ethylene glycol as a killing-preserving solution. Traps were covered with a 20 cm × 20 cm piece of fiberboard to protect them from litter and rain. Trapped ground beetles were collected every 2 weeks from April to October, 2001. All ground beetles were identified to species using keys in Hůrka^39^. Only continuous sampling covering the whole beetle activity period provides a reliable relative measure of co-occurring ground beetle species’ abundances^40^, which is crucial to evaluate accurately community assembly mechanisms. For evaluation, therefore, pitfall trap catches of each site were combined for the whole sampling period (from April to October), resulting in 12 data sets.

### Data analyses

During the evaluation of functional features, traits related to morphology, reproduction, dispersal and resource use were applied (Table 1) using published data^39,41–43^. Distances between species based on functional traits (FDist) were calculated by Gower’s distance metric, using the *StatMatch* package^44^. Phylogenetic (evolutionary) distance (PDist) was characterized by the distance between species based on the branch length to the common ancestor on Beutel *et al*.’s^45^ phylogenetic tree. To assess conservatism or convergence of the studied functional traits, the relationship between functional and phylogenetic distances was evaluated by Mantel-tests using the *ade4* package with 9999 replications^46^.

**Table 1:** Traits used to calculate functional distances between ground beetle species.

Trait	Type/unit	Trait range or category	Relevance/ecological meaning
Body size	Continuous/mm	3.4–28	It correlates with species physiology and life history
Wing morphology	Categorical	brachypterous/dimorphic/macropterous	It is linked to dispersal capacity and recolonization potential
Overwintering type	Ordinal	summer larvae/winter larvae/flexible	It is correlated with seasonality and linked to natural and/or human disturbance regimes
Daily activity	Categorical	diurnal/nocturnal	Species having similar seasonality and habitat use can be separated by their temporal niches
Diet	Categorical	herbivorous*/mixed feeder/predator	Species having similar habitat use can differ in their feeding strategies
Habitat affinity	Ordinal	forest/generalist/open-habitat	Generalist and open-habitat species are often the first arrivals after disturbances in forests
Humidity preference	Ordinal	xerophilous/mesophilous/hygrophilous	Important preference trait in forests for niche separation

^*^Including both granivorous and frugivorous feeding strategies^86^.

Recently, Cadotte *et al*.^16^ suggested that during the consideration of similarity among species, not only the selected traits but phylogenetically correlated traits should also be considered, since phylogeny may provide additional and/or complementary information to functional traits. Distances between species in trait-space and between species in the phylogenetic-space could be combined as functional-phylogenetic distance (*FPDist*):

$$FPDist={(aPDis{t}^{p}+(1-a)FDis{t}^{p})}^{1/p},$$

where *PDist* is the phylogenetic distance; *FDist* is the functional distance, *p* is an integer (*p* = 2 is the Euclidean distance, recommended by Cadotte *et al*.^16^), while *a* is the weighting parameter, which determines the contribution of *PDist* and *FDist* to *FPDist*.

To evaluate community assembly mechanisms, we first calculated functional-phylogenetic distance for 41 levels of the functional-phylogenetic weighting parameter (*a*) from 0 to 1 by increasing steps of 0.025. Next, standardized effect sizes were calculated for all 41 levels, using the observed mean pairwise functional-phylogenetic distance of all species pairs collected in each sampling sites and the same of randomly selected species^16,20,47^. Ground beetles are good colonizers by walking and/or flying^30^; therefore, we assumed that each species is able to colonize a given site with equal probability. Based on this assumption, during the construction of null assemblages, the species were chosen randomly without replacement from the regional species pool, so the species richness of each site and the total abundance of all species across all sites were kept constant. To decrease the influence of rare and possibly “erratic” species, the distance values were weighted by species abundance^48^. The standardized effect sizes were calculated based on null models with 999 randomizations using the *picante* package^49^. Third, the optimal value of the weighting parameter (*a*) was determined. The strength of the relationship between the standardized effect sizes and the position along the urbanization gradient was tested by systematically changing the phylogenetic-weighting parameter (*a*) using linear models with the function *lm*. The optimal value of the weighting parameter (*a*) was at the maximum of the adjusted *R*^2^ value of the linear model between the standardized effect sizes and the position along the urbanization gradient^16,20^. Finally, we evaluated the mean standardized effect sizes calculated for the optimal value of the weighting parameter (*a*). A mean standardized effect size was considered to be significantly different from zero if its respective confidence interval did not include zero. Confidence intervals were calculated using the *boot* package with 999 iterations. Significant negative values of the standardized effect sizes indicate functional and phylogenetic clustering, a sign of environmental filtering, while significant positive values indicate repulsion, resulting from species interactions. If communities are stochastically structured, the standardized effect sizes do not differ significantly from zero^17,26^. The R program environment (version 3.4.3^50^) was used during the analyses.

## Results

The sampling effort of 21 840 trap-days (120 traps × 182 days) resulted in 2140 individuals of 50 ground beetle species. There were 25 species with 1206 individuals in the rural sites, 457 individuals of 26 species in the suburban sites, and 43 species with 477 individuals in the urban sites. Colonisation of matrix species (open-habitat and generalist ones) from the surrounding agricultural landscapes is the main reason for the higher species richness in urban sites compared to rural ones. In the urban sites, trapped species were predominantly matrix species (41 species; Supplementary Material, Table S1).

**Figure 1:**
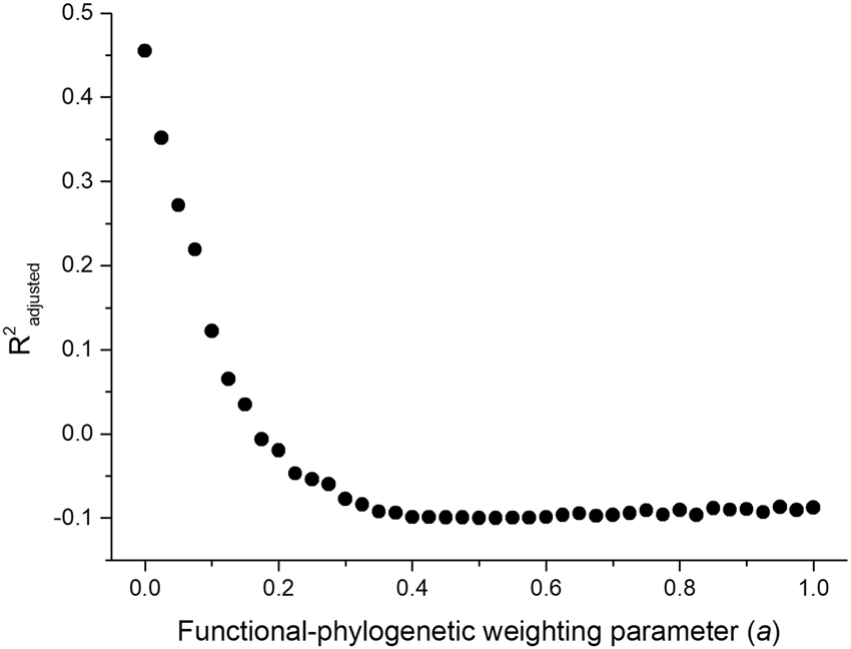
The strength of the relationship (expressed as *R*^*2*^_*adjusted*_) between the standardized effect size and the position along the urbanization gradient for 41 levels of the functional-phylogenetic weighting parameter (*a*).

**Figure 2:**
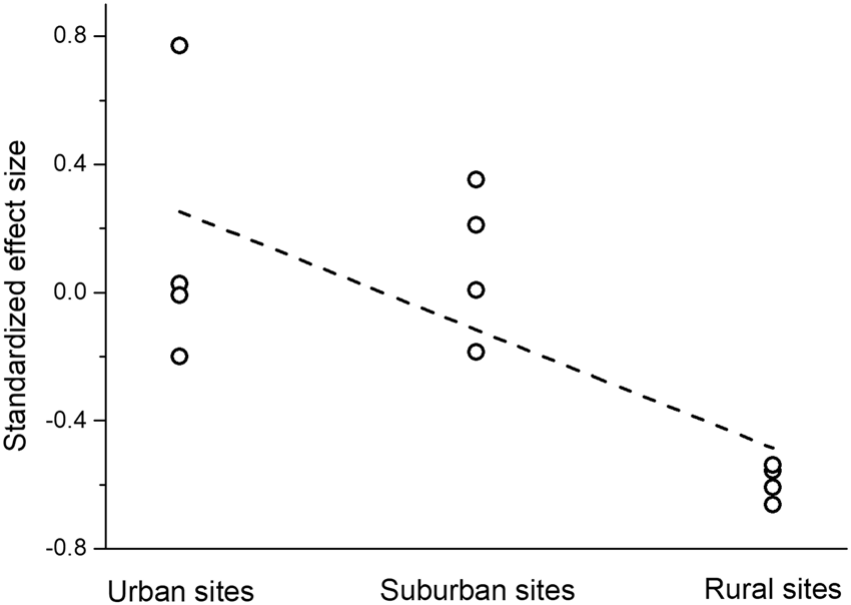
The significant negative relationship (*F* = 10.9891, *d.f*. = 1, 10, *p* = 0.0078) between the standardized effect sizes and the position along the urbanization gradient at the phylogenetic-weighting parameter of *a* = 0.

The functional (trait-based) and phylogenetic pairwise distance values were significantly correlated (Mantel-test, *R* = 0.1818, *p* < 0.001), indicating that the more closely related species have more similar traits (trait conservatism). Standardized effect sizes were positively correlated with the position along the rural-suburban-urban gradient for all values of the phylogenetic-weighting parameter (*a*), showing that environmental filtering in the rural sites strongly selected for communities of functionally and phylogenetically similar species. The variance explained by the position along the gradient was maximized at *a* = 0 (maximal adjusted *R*^2^ = 0.4550, Figs 1 and 2), after which it decreased continuously (Fig. 1). This suggests that functional information considered in the present situation was more important than phylogenetic one to reveal assembly rules, likely due to the confirmed trait conservatism.

**Figure 3:**
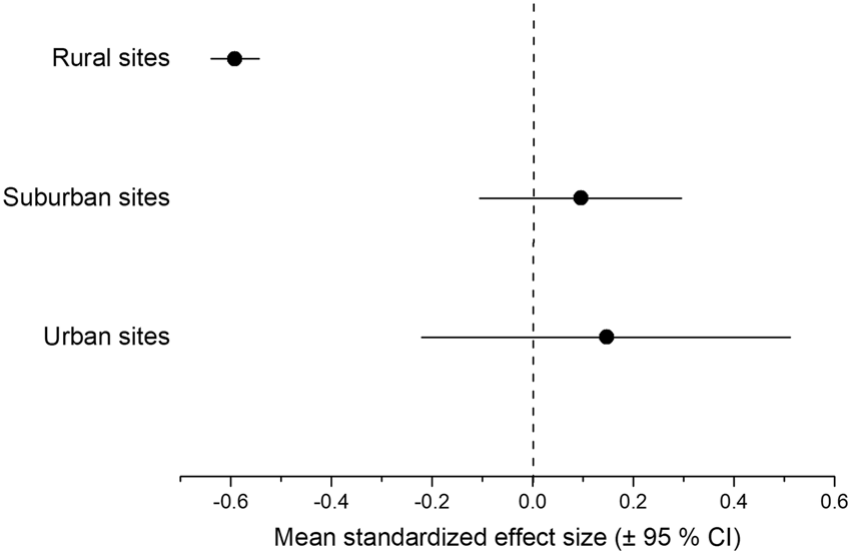
The mean standardized effect sizes calculated for phylogenetic-weighting parameter value of *a* = 0 (±95% confidence interval) along the urbanization gradient.

At the optimal value of the weighting parameter (*a* = 0) ground beetle communities in the rural sites showed significant functional and phylogenetic clustering (under-dispersion), indicating that co-existing species were functionally and phylogenetically more similar than expected by chance (Fig. 3). The mean standardized effect sizes for urban and suburban assemblages, calculated for *a* = 0, were not significantly different from zero, indicating a stochastically (randomly) structured ground beetle assemblage (Fig. 3).

## Discussion

Co-occurring ground beetle species in the studied rural sites were more closely related and functionally more similar than expected by chance, indicating environmental filtering. It is likely that the key filter conditions of stable, undisturbed rural sites are abiotic environmental parameters, such as high humidity, low soil, and soil surface temperature. Humidity and temperature are among the most important environmental factors generally influencing spatial distribution and persistence of ground beetles, and particularly the larvae that have limited mobility, weak chitinization, and therefore narrower tolerance limits than adults do^20,30^. Another key factor for filtering co-existing species in the rural sites could be the structural habitat variables (cover and thickness of leaf litter layer, amount of coarse woody debris, cover of herbs, shrubs, and canopy). Complex and heterogeneous habitats offer more food items, microhabitats, and shelter from adverse microclimatic conditions^51–53^, thereby may strongly influence the composition of ground beetle assemblages^54,55^. Food conditions also constitute a potential environmental filter, as colonization, survival and persistence of ground beetles in a habitat are crucially determined by available food, be that invertebrates, seeds, or plant tissues^30^. Previous studies conducted in the same sites^33,36,56^ proved that the abundance and species composition of ground beetles were significantly influenced by the above conditions leading to communities composed of predominantly carnivorous forest species^33^. Based on our results, these environmental filters are selecting for related forest species with specific, phylogenetically conserved traits that allow to cope with rural habitat conditions, resulting in functional and phylogenetic clustering.

Our findings suggest that the environmental conditions in undisturbed forests produced strong environmental filtering, leading to communities composed of species with phylogenetically conserved functional traits that enable more effective habitat exploitation under these conditions^20^. Likewise, related and functionally similar ground beetle species^20^ and closely related woody plant species^57^ are filtered by environmental factors in undisturbed forests. Environmental filtering is also the main assembly rule of nonvolant small mammals in southern Brazilian forests, where closely related and ecologically similar species coexist^58^. Bird assemblages from Amazonian pristine forest also tend to be functionally clustered, indicating environmental filtering^59^.

Recently, it was emphasized that environmental filtering is not the only force leading the co-existence of closely related and/or ecologically similar species^26,60,61^. Vamosi & Vamosi^62^, studying predaceous diving beetles, suggested that predation risk may produce phylogenetic clustering, as effective defensive traits against a particular predator may be phylogenetically conserved. Differences in competitive ability (termed also fitness differences) among co-occurring species (e.g. differences in ability to deplete limiting resources, differences in susceptibility to predation, variation in the number of viable offspring) can drive competitive exclusion^63^. Therefore, competition can also produce assemblages with more similar and more related taxa, even when the traits underlying the competitive ability differences are phylogenetically conserved^63^. Moreover, indirect biotic interaction (pollinator-mediated facilitation) may also generate phylogenetic and/or functional clustering^64^. These underline that environmental filtering and biotic interactions should be simultaneously considered when evaluating community assembly mechanisms^26^. Environmental filtering and biotic interactions act together to create assembly rules structuring communities: environmental filters are likely to be the first restriction permitting only species with appropriate traits for the target environment to establish populations, but even species that passed the environmental filters could be filtered out by a second filter, composed of biotic interactions^25,26,63^.

In community organization of ground beetles, competition (interspecific and intraguild competition), interaction with pathogens, parasites, and predators could be the most important biotic interactions^30^. The importance of interspecific competition in ground beetle community organization is inconclusive, because most experimental, manipulative studies suffer from methodological limitations (unrealistic densities, non-comparable methods and habitats used in the studies) as it was highlighted previously^30,65^. Significant interspecific competition was found in the USA^66,67^, but not in Belgium^68^. Barraclough *et al*.^69^, studying resource-partitioning between co-occurring tiger beetle species, found no evidence of species interactions influencing community organization. However, food limitation under field conditions for both larval^70^ and adult ground beetles^66,71^ suggest that interspecific competition for food could influence community assembly in ground beetles. Moreover, most studies on competition among ground beetle species focused on the adult stages, but larvae have more restricted food spectrum, and are less adapted to tolerate food shortages. Accordingly, the importance of interactions between adults and larvae or between larvae may be more important than between adults^30^. Furthermore, strong intra-guild competition exists between ground beetles and other ground-dwelling generalist predators, like spiders and ants^30^. For these reasons, it seems that competition has a role in community organization of ground beetles, but the strength of competition may be weak compared to environmental filtering^69^. In urban sites, supplemental food resources by humans (e.g. pet food and garbage)^72^ can increase the amount of potential food items for omnivorous and carnivorous ground beetle species. Moreover, in the studied situation, abundance of ground beetles was significantly lower in urban sites compared to rural ones^33^. In the urban sites the presence of alternative food resources and the lower abundance of beetles can also reduce the strength of both the interspecific and intraguild competition. Based on the above, it is assumed that competition may have a role in community assembly of ground beetles in rural sites more than in urban ones. Pathogens and parasites cause significant mortality for all ground beetle developmental stages^30^. Mortality of *Pterostichus oblongopunctatus* eggs incubated in fresh litter was 83%, while after eliminating other organisms from litter by heating, the egg mortality fell to 18%^73^. Under field conditions, parasitism can cause larval mortality up to 25%, while parasitic nematodes and ectoparasitic fungi are present on up to 41% of adult ground beetles^30^. Changes in environmental conditions caused by urbanization (e.g., increasing mean temperature^7^, decreasing humidity^8^) may also negatively affect the density and prevalence of pathogens and parasites^1^, and their infections. Indeed, significantly higher prevalence and intensity of mites on a ground beetle species was found in rural sites than in urban ones^74^. Therefore, pathogens and parasites, as biological filters, may have more influence on ground beetle community organization in rural sites compared to urban ones, as closely related species may be similarly susceptible to infection^75^. Most observations also indicated that predation by vertebrates (birds, mammals, especially small mammals) is an important mortality factor for adult ground beetles^30^. Predation pressure, however, seems to decrease with increasing urbanization^72^, therefore predation may have effect on the organization of ground beetle assemblages more in the rural sites than in urban ones, as closely related species may have similar defensive traits to avoid predation^62^,

In the studied region urbanization caused pronounced changes in environmental and habitat characteristics^33,56^. Temperature both in the soil and on the soil surface were significantly higher, while humidity was significantly lower at the urban sites compared to the rural ones^56^. Maintenance operations in urban sites altered the structural habitat variables, since the amount of coarse woody debris and the cover of herbs were significantly lower here than in the rural sites^56^. All these changes caused by urbanization in environmental parameters and structural habitat variables appeared to eliminate the combination of factors necessary for forest specialist species^76^, and contributed to their decline^33,36,56,77^. These changes have significant impact on other ground-dwelling invertebrates (potential prey items for ground beetles), and their numbers also drastically decreased in urban sites compared to rural ones^56,78^. The impoverishment of food spectrum may also contribute to the decline of predominantly predator forest specialist species. Moreover, reduced dispersal and gene flow of forest specialist species between fragmented and isolated natural forest patches in urbanized areas may lead to reduced genetic diversity and higher population extinction probability as a consequence of lower genetic diversity^79^. Recent studies reviewing and/or re-analysing ground beetle data^80–82^ found a consistent, pronounced decrease of forest specialist species in urban forest fragments compared to their rural counterparts. Changes in environmental parameters and structural habitat variables driven by urbanization create microhabitats that are significantly different from those in undisturbed forest floor habitats. Matrix species (open-habitat and generalist ones) from the surrounding agricultural landscapes can easily colonize these microhabitats that are typically warm, with a dry microclimate and open canopy^33,34^, and their density usually increases at urban sites^82^. It seems, however, that the success of such colonization events in urban sites is unpredictable. Such unpredictable, random species flow from the surrounding matrix^36^ may lead to stochastically (randomly) structured ground beetle assemblages. Co-occurring ground beetle species in suburban sites were also functionally and phylogenetically randomly structured. This suggests that the limited changes at suburban sites also encourage the random influx of ground beetles from the surrounding matrix. Similarly to our results, Kitagawa *et al*.^83^, studying assembly processes of understorey woody communities, found that modification of habitat structure by thinning brought about an increase in the relative importance of stochastic immigration. Studies on other taxa also emphasized that dispersal processes promote the appearance of randomly structured assemblages (for birds^59,84^; for beetles^20^).

Our results, which showed that urbanization lead to randomly structured ground beetle assemblages at our urban and suburban sites, also have an important conservation message. The widespread practices used in urbanized areas (strong thinning, removing decaying wood material and cut plant biomass, creating asphalt-covered paths) cause pronounced changes in environmental and habitat characteristics, contributing to the significant decline of forest specialist species and significant increase of matrix species^33^. Therefore, there is a growing need for appropriate management strategies which simultaneously consider recreational, economic and conservation criteria to conserve/restore forest specialist species adapted to live in forested ecosystem^85^. Management practices which minimize the modification of habitat characteristics and try to mimic natural processes could serve both the demands of city dwellers and the maintenance of naturally structured biotic communities.

## Conclusion

Earlier studies demonstrate that taxonomic diversity and species composition of ground beetle markedly differ along the urbanization gradient, leading to a decrease in specialist species^80–82^. Our study, in addition, indicated that different drivers were active behind these patterns. Understanding such patterns, by simultaneously considering functional and phylogenetic information, helps to predict the effects of human activities on the community assembly processes, as well as on ecosystem functioning^24^. Our results indicated that urbanization lead to a decline in the strength of environmental filtering, and suburban and urban habitats were randomly colonized by species from the surrounding matrix. Based on our findings it is important to stress that more attention to manage urban green spaces to maintain conditions allowing the continued functioning of environmental filters may help to retain more native biodiversity and proper ecosystem functioning, with its benefits for human inhabitants of urbanized areas.

## Electronic supplementary material

Supplementary information
