# Supplementary material for: Conversion from environmental filtering to randomness as assembly rule of ground beetle assemblages along an urbanization gradient
Source: Sci Rep. 2018 Nov 19;8:16992. doi: 10.1038/s41598-018-35293-8 (PMC6242958; doi:10.1038/s41598-018-35293-8)
Supplement: Supplementary file 1 — Supplementary information [file 41598_2018_35293_MOESM1_ESM.docx]

**SUPPLEMENTARY INFORMATION**

Magura T, Lövei GL, and Tóthmérész B.

Conversion from environmental filtering to randomness as assembly rule of ground beetle assemblages along an urbanization gradient. *Scientific Reports*

**Table S1.** Life-history traits (body size, wing morphology, mode of overwintering, daily activity and diet) and ecological traits (habitat affinity and humidity preference) of the trapped ground beetles and their abundance across the studied urbanization gradient.

| **Species** | **Body size, mm** | **Wing morphology** | **Over-wintering as** | **Daily activity** | **Diet** | **Habitat preference** | **Humidity preference** | **Abundance (no. ind.) in** | | |
| --- | --- | --- | --- | --- | --- | --- | --- | --- | --- | --- |
|  |  |  |  |  |  |  |  | **Urban** | **Sub-urban** | **Rural** |
| *Agonum lugens* | 9 | macropterous | adult | nocturnal | predatory | generalist | hygrophilous | 1 | 0 | 0 |
| *Amara anthobia* | 6.2 | macropterous | adult | diurnal | omnivorous | generalist | xerophilous | 5 | 0 | 0 |
| *A. communis* | 6.5 | macropterous | adult | diurnal | herbivorous | open-habitat | mesophilous | 5 | 0 | 0 |
| *A. consularis* | 8.3 | macropterous | larva | nocturnal | omnivorous | open-habitat | mesophilous | 0 | 1 | 0 |
| *A. convexior* | 7.7 | macropterous | adult | diurnal | omnivorous | generalist | xerophilous | 52 | 6 | 12 |
| *A. familiaris* | 6.3 | macropterous | adult | diurnal | herbivorous | generalist | mesophilous | 4 | 3 | 4 |
| *A. ovata* | 8.6 | macropterous | adult | diurnal | herbivorous | generalist | xerophilous | 3 | 0 | 0 |
| *A. saphyrea* | 8.8 | macropterous | adult | nocturnal | herbivorous | forest | hygrophilous | 4 | 8 | 26 |
| *A. similata* | 8.6 | macropterous | adult | diurnal | herbivorous | open-habitat | mesophilous | 2 | 1 | 1 |
| *Anchomenus dorsalis* | 6.6 | macropterous | adult | nocturnal | omnivorous | open-habitat | mesophilous | 1 | 0 | 0 |
| *Anisodactylus nemorivagus* | 8.9 | macropterous | adult | nocturnal | omnivorous | open-habitat | hygrophilous | 26 | 0 | 0 |
| *Asaphidion flavipes* | 4.3 | macropterous | adult | diurnal | predatory | open-habitat | mesophilous | 2 | 0 | 0 |
| *Badister bullatus* | 5.2 | macropterous | adult | nocturnal | predatory | generalist | mesophilous | 4 | 0 | 0 |
| *B. lacertosus* | 6.3 | macropterous | adult | nocturnal | predatory | generalist | hygrophilous | 2 | 9 | 1 |
| *B. meridionalis* | 6.7 | macropterous | adult | nocturnal | predatory | generalist | hygrophilous | 7 | 2 | 0 |
| *Bembidion lampros* | 3.4 | dimorphic | adult | diurnal | predatory | open-habitat | mesophilous | 29 | 0 | 3 |
| *Calathus erratus* | 9.5 | dimorphic | larva | nocturnal | predatory | generalist | xerophilous | 1 | 0 | 0 |
| *C. fuscipes* | 11.1 | dimorphic | larva | nocturnal | omnivorous | open-habitat | xerophilous | 11 | 0 | 0 |
| *C. melanocephalus* | 7.1 | dimorphic | larva | nocturnal | omnivorous | generalist | xerophilous | 1 | 0 | 0 |
| *Carabus convexus* | 17 | macropterous | adult | nocturnal | predatory | forest | xerophilous | 0 | 41 | 46 |
| *C. granulatus* | 19.5 | dimorphic | adult | nocturnal | predatory | generalist | hygrophilous | 2 | 1 | 1 |
| *C. ullrichi* | 27 | macropterous | adult | nocturnal | predatory | generalist | mesophilous | 1 | 0 | 0 |
| *C. violaceus* | 28 | macropterous | adult | nocturnal | predatory | generalist | mesophilous | 51 | 30 | 124 |
| *Clivina fossor* | 5.9 | dimorphic | adult | nocturnal | omnivorous | generalist | hygrophilous | 3 | 0 | 0 |
| *Diachromus germanus* | 8.4 | macropterous | adult | diurnal | herbivorous | open-habitat | hygrophilous | 1 | 0 | 0 |
| *Harpalus distinguendus* | 9.5 | macropterous | adult/larva | diurnal | omnivorous | open-habitat | xerophilous | 0 | 1 | 0 |
| *H. latus* | 9.1 | macropterous | adult/larva | diurnal | omnivorous | generalist | hygrophilous | 6 | 0 | 9 |
| *H. luteicornis* | 7.1 | macropterous | adult | diurnal | omnivorous | generalist | xerophilous | 5 | 20 | 1 |
| *H. tardus* | 9.4 | macropterous | adult | diurnal | omnivorous | open-habitat | xerophilous | 34 | 53 | 35 |
| *Leistus ferrugineus* | 6.8 | macropterous | larva | nocturnal | predatory | generalist | mesophilous | 0 | 0 | 1 |
| *Licinus depressus* | 10.4 | macropterous | adult | nocturnal | predatory | open-habitat | xerophilous | 6 | 0 | 0 |
| *Notiophilus biguttatus* | 4.9 | dimorphic | adult | diurnal | predatory | generalist | mesophilous | 2 | 0 | 0 |
| *N. palustris* | 5.1 | dimorphic | adult | diurnal | predatory | generalist | hygrophilous | 5 | 1 | 2 |
| *N. rufipes* | 5.3 | dimorphic | adult | diurnal | predatory | generalist | mesophilous | 28 | 11 | 3 |
| *Ophonus nitidulus* | 9.6 | dimorphic | adult | diurnal | herbivorous | generalist | xerophilous | 1 | 1 | 32 |
| *Ophonus schaubergerianus* | 8.8 | macropterous | adult | diurnal | herbivorous | open-habitat | xerophilous | 0 | 0 | 1 |
| *Oxypselaphus obscurus* | 5.5 | dimorphic | adult/larva | nocturnal | predatory | generalist | hygrophilous | 0 | 1 | 1 |
| *Panagaeus bipustulatus* | 7.2 | macropterous | adult | diurnal | omnivorous | generalist | hygrophilous | 4 | 0 | 0 |
| *Platyderus rufus* | 6.3 | macropterous | larva | nocturnal | predatory | generalist | mesophilous | 45 | 18 | 40 |
| *Poecilus cupreus* | 11.8 | macropterous | adult | diurnal | omnivorous | open-habitat | hygrophilous | 1 | 0 | 0 |
| *Pseudoophonus rufipes* | 13.1 | macropterous | adult/larva | nocturnal | omnivorous | open-habitat | xerophilous | 6 | 4 | 12 |
| *Pterostichus macer* | 12.9 | macropterous | adult | nocturnal | omnivorous | open-habitat | mesophilous | 1 | 0 | 0 |
| *P. melanarius* | 15.7 | dimorphic | larva | nocturnal | predatory | generalist | mesophilous | 33 | 1 | 0 |
| *P. melas* | 14.9 | macropterous | adult | nocturnal | predatory | generalist | mesophilous | 1 | 0 | 0 |
| *P. niger* | 18.4 | macropterous | larva | nocturnal | predatory | generalist | hygrophilous | 9 | 4 | 17 |
| *P. oblongopunctatus* | 11.5 | macropterous | adult | nocturnal | predatory | forest | mesophilous | 60 | 197 | 795 |
| *P. strenuus* | 6 | dimorphic | adult | nocturnal | omnivorous | generalist | hygrophilous | 10 | 25 | 7 |
| *Stomis pumicatus* | 7.5 | brachypterous | larva | nocturnal | predatory | generalist | hygrophilous | 1 | 14 | 19 |
| *Synuchus vivalis* | 7.2 | dimorphic | larva | nocturnal | omnivorous | open-habitat | mesophilous | 1 | 3 | 13 |
| *Trechus quadristriatus* | 3.5 | dimorphic | adult/larva | nocturnal | predatory | generalist | mesophilous | 0 | 1 | 0 |
